# Ensemble Entropy with Adaptive Deep Fusion for Short-Term Power Load Forecasting

**DOI:** 10.3390/e28020158

**Published:** 2026-01-31

**Authors:** Yiling Wang, Yan Niu, Xuejun Li, Xianglong Dai, Xiaopeng Wang, Yong Jiang, Chenghu He, Li Zhou

**Affiliations:** 1 College of Computer Science and Technology, Taiyuan University of Technology, Taiyuan 030024, China; 2023002052@link.tyut.edu.cn (Y.W.); niuyan@tyut.edu.cn (Y.N.); 2 Jiangsu Haohan Information Technology Co., Ltd., Nantong 226300, China; xuejun.li@haohanit.com.cn (X.L.); xianglong.dai@haohanit.com.cn (X.D.); xiaopeng.wang@haohanit.com.cn (X.W.); jiang.yong@haohanit.com.cn (Y.J.); tiger.he@haohanit.com.cn (C.H.)

**Keywords:** power load forecasting, multimodal time series analysis, ensemble entropy, deep learning, LSTM, adaptive fusion

## Abstract

Accurate power load forecasting is crucial for ensuring the safety and economic operation of power systems. However, the complex, non-stationary, and heterogeneous nature of power load data presents significant challenges for traditional prediction methods, particularly in capturing instantaneous dynamics and effectively fusing multi-feature information. This paper proposes a novel framework—Ensemble Entropy with Adaptive Deep Fusion (EEADF)—for short-term multi-feature power load forecasting. The framework introduces an ensemble instantaneous entropy extraction module to compute and fuse multiple entropy types (approximate, sample, and permutation entropies) in real-time within sliding windows, creating a sensitive representation of system states. A task-adaptive hierarchical fusion mechanism is employed to balance computational efficiency and model expressivity. For time-series forecasting tasks with relatively structured patterns, feature concatenation fusion is used that directly combines LSTM sequence features with multimodal entropy features. For complex multimodal understanding tasks requiring nuanced cross-modal interactions, multi-head self-attention fusion is implemented that dynamically weights feature importance based on contextual relevance. A dual-branch deep learning model is constructed that processes both raw sequences (via LSTM) and extracted entropy features (via MLP) in parallel. Extensive experiments on a carefully designed simulated multimodal dataset demonstrate the framework’s robustness in recognizing diverse dynamic patterns, achieving MSE of 0.0125, MAE of 0.0794, and R^2^ of 0.9932. Validation on the real-world ETDataset for power load forecasting confirms that the proposed method significantly outperforms baseline models (LSTM, TCN, transformer, and informer) and traditional entropy methods across standard evaluation metrics (MSE, MAE, RMSE, MAPE, and R^2^). Ablation studies further verify the critical roles of both the entropy features and the fusion mechanism.

## 1. Introduction

Accurate short-term power load forecasting (STLF) is essential for ensuring grid reliability, economic dispatch, and renewable energy integration in modern power systems [1,2]. With the rapid development of smart grids and advanced metering infrastructure, precise predictions have become increasingly important for real-time dispatch, demand response, and energy market operations [3,4].

Traditional STLF methods, including statistical models (e.g., ARIMA) and machine learning approaches (e.g., SVR, RF), often struggle with the non-stationary, nonlinear, and multimodal nature of real-world load data [5]. Deep learning models, particularly Long Short-Term Memory (LSTM) networks [6], Gated Recurrent Units (GRUs) [7], and Temporal Convolutional Networks (TCNs) [8], have demonstrated superior capability in capturing temporal dependencies. More recently, transformer-based architectures [9] and their variants like Informer have shown state-of-the-art performance in long sequence forecasting. However, these methods typically focus on temporal patterns and may overlook the intricate, instantaneous complexity dynamics and cross-modal interactions inherent in integrated energy systems [10]. Recent studies on multimodal data fusion [11,12] and multi-spatiotemporal feature integration highlight the need for more sophisticated approaches that can effectively capture cross-modal interactions and adapt to varying signal characteristics [13].

Despite these advancements, contemporary STLF research continues to face several critical challenges that limit practical application. First, while deep learning models have demonstrated impressive predictive accuracy, they often operate as black boxes, making it difficult to interpret the relationship between input features and forecasting outputs. Recent work by [10] attempted to address this limitation through Kolmogorov–Arnold Networks (KANs), which provide interpretable analytical expressions for STLF. However, such interpretable models may sacrifice some predictive accuracy and struggle with highly complex, multimodal data patterns. Second, the extraction of spatiotemporal features from load data remains challenging. Methods like the multi-scale graph tide model proposed by [11] attempt to enhance graph structure learning and spatiotemporal decoupling, but they require predefined spatial relationships and may not adapt well to systems with rapidly changing topologies. Third, data privacy concerns in distributed systems have led to the development of privacy-preserving approaches. The federated model-agnostic meta learning (FMAML) method introduced by [12] addresses data isolation issues through federated learning, but such methods can suffer from slow convergence and reduced accuracy when they are applied to highly heterogeneous client data. Notably, emerging technologies like quantum computing have also started to be applied to power system analytics. For instance, ref. [14] proposed a quantum kernel embedding method that utilizes shallow, fixed-depth circuits to generate highly expressive feature maps for detecting false data injection attacks in power systems. This type of approach demonstrates the potential of quantum machine learning in solving complex power system problems, although computational cost and task-specific design remain key research foci.

Beyond these architectural challenges, a fundamental limitation shared by many current STLF approaches is their inadequate handling of signal complexity dynamics. Power load data exhibit rich nonlinear characteristics, transient behaviors, and multi-scale patterns that are not fully captured by models focusing solely on temporal dependencies. Entropy, as a measure of system complexity and uncertainty, has been introduced to power load analysis to quantify signal irregularity [13,15]. Established entropy measures, such as approximate [16], sample [17], and permutation [18], have been applied to power load forecasting. Nevertheless, these entropy-based approaches face significant limitations in the context of STLF: (1) They often compute global entropy over entire time series, losing sensitivity to instantaneous local dynamics critical for short-term forecasting. (2) They are typically applied to single modalities, lacking an effective mechanism to fuse multi-feature information. (3) Their integration with deep temporal models is often simplistic, failing to adaptively balance feature contributions [19,20]. Recent advances in probabilistic multi-energy forecasting frameworks, such as attentive quantile regression temporal convolutional networks [21], further highlight the importance of capturing both correlations between different loads and their inherent uncertainties.

To address these limitations, this paper proposes a novel framework—Ensemble Entropy with Adaptive Deep Fusion (EEADF)—for short-term multi-feature power load forecasting. The framework introduces three key innovations that collectively address the shortcomings of existing methods: (1) An ensemble instantaneous entropy module that extracts and fuses multiple entropy types (ApEn, SampEn, and PermEn) in parallel within sliding windows, providing a real-time, multi-scale representation of local complexity for each feature. (2) A task-adaptive hierarchical fusion mechanism that balances computational efficiency and model expressivity by dynamically selecting between feature concatenation and multi-head self-attention based on task requirements. (3) A dual-branch architecture that simultaneously processes raw temporal sequences (via LSTM) and entropy-derived complexity features (via MLP), enabling comprehensive modeling of both temporal dependencies and instantaneous dynamics. These innovations work synergistically to overcome the limitations of existing approaches in capturing local dynamic patterns and effectively fusing multi-modal information.

The main contributions of this work are as follows:

1. An ensemble instantaneous entropy module that extracts and fuses multiple entropy types (ApEn, SampEn, and PermEn) in parallel within sliding windows, providing a real-time, multi-scale representation of local complexity for each feature.
2. A task-adaptive hierarchical fusion mechanism that balances computational efficiency and model expressivity by employing feature concatenation for structured forecasting tasks and multi-head self-attention for complex multimodal understanding tasks.
3. A dual-branch deep architecture that processes raw temporal sequences (via LSTM) and entropy-derived complexity features (via MLP) in parallel, then fuses them adaptively, leveraging both temporal dependencies and instantaneous dynamics.
4. Extensive experiments on both a newly designed simulated multimodal dataset and the public ETDataset validate the framework’s superiority over baseline models (LSTM, TCN, transformer, informer) and traditional entropy methods using standard metrics (MSE, MAE, RMSE, MAPE, and R^2^). Ablation studies confirm the critical role of each component.

## 2. Methodology

**Figure 1:**
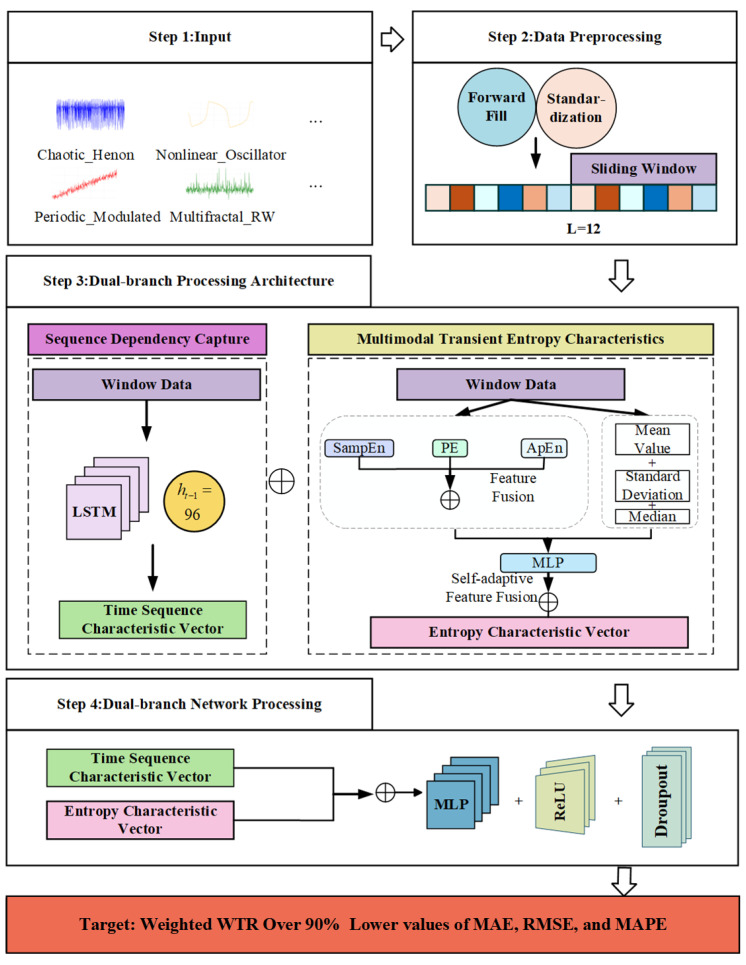
Framework of the ensemble entropy-based adaptive deep fusion model for short-term load forecasting.

Figure 1 illustrates the complete computational pipeline of the proposed framework for power load forecasting. The process begins with raw multi-feature power load data, including temporal, meteorological, and pricing information. The data first undergo preprocessing and are segmented using a unified sliding window. For each window, the framework employs a dual-branch processing architecture: one branch extracts multimodal instantaneous entropy features and statistical features from these window data. The other branch feeds the raw sequence of the same window into an LSTM network to capture temporal dependencies. Finally, the outputs from both branches are summed and fed into a prediction network to generate the final result. The specific computational steps are elaborated in the following subsections.

### 2.1. Signal Preprocessing and Instantaneous Segmentation

Given a power load dataset containing multiple features, such as moment, temperature, humidity, and electricity price, let the total number of features be *D*, where the target variable is the power load *y*, and the rest are feature variables. The observation vector at time point *t* is denoted as $X_{t}=\left\{x_{t}^{\left(1\right)},x_{t}^{\left(2\right)},\ldots ,x_{t}^{\left(D\right)}\right\}$.

First, perform forward filling to handle missing values:

(1) $$x_{t}^{\left(d\right)}=\left\{\begin{array}{cc} x_{t}^{\left(d\right)} & \;\mathrm{if}\;\mathrm{not}\;\mathrm{NaN}\\ x_{t-1}^{\left(d\right)} & \;\mathrm{if}\;\mathrm{NaN}\;\mathrm{and}\;t>1.\\ 0 & \;\mathrm{otherwise}\; \end{array}\right.$$

Subsequently, use a sliding window of length *L* to segment the sequence. For any time point t(t≥L), its corresponding local window data are defined as the following:

(2) $$W_{t}={\left[X_{t-L+1},X_{t-L+2},\ldots ,X_{t}\right]}^{T}$$

where $W_{t}\in R^{L\times D}$. This window $W_{t}$ is used for both instantaneous entropy calculation and sequence prediction.

### 2.2. Multimodal Instantaneous Entropy Feature Extraction

The instantaneous entropy for each feature dimension is calculated within each sliding window $W_{t}$ to capture the local dynamic complexity of each power signal feature, addressing the non-stationarity and cross-interference issues in power load data. Based on the code implementation, three types of entropy are calculated as discussed below.

#### 2.2.1. Approximate Entropy (ApEn) [16]

Approximate entropy is used to measure the regularity of a time series [22,23,24]. For a univariate window sequence U={u(1),u(2),…,u(L)} (from a specific feature column of $W_{t}$), the calculation steps are as follows:

Construct m-dimensional vectors:

(3) X(i)={u(i),u(i+1),…,u(i+m−1)},i=1,2,…,L−m+1.

Define the distance between vectors:

(4) $$d\left[X\left(i\right),X\left(j\right)\right]=\max_{k=0,\ldots ,m-1}\left|u\left(i+k\right)-u\left(j+k\right)\right|.$$

For each *i*, count the number of instances where d[X(i),X(j)]≤r and calculate:

(5) $$C_{i}^{m}\left(r\right)=\frac{\mathrm{count}}{L-m+1}$$

(6) $$\Phi^{m}\left(r\right)=\frac{1}{L-m+1}\sum_{i=1}^{L-m+1}\ln C_{i}^{m}\left(r\right).$$

Approximate entropy is defined as follows:

(7) $$ApEn\left(m,r\right)=\Phi^{m}\left(r\right)-\Phi^{m+1}\left(r\right),\;m=2,r=0.2\times \mathrm{std}\left(U\right).$$

#### 2.2.2. Sample Entropy (SampEn) [17]

Sample entropy is an improvement over Approximate Entropy, offering better consistency [22,23]. The calculation process is similar to approximate entropy but excludes self-matches during template matching:

Construct *m*-dimensional vectors X(i) and m+1-dimensional vectors Y(i) and calculate the match counts for dimensions *m* and m+1:

(8) $$B=\sum_{i=1}^{L-m}\sum_{j=1,j\neq i}^{L-m}I\left[d\left[X\left(i\right),X\left(j\right)\right]\leq r\right]$$

(9) $$A=\sum_{i=1}^{L-m}\sum_{j=1,j\neq i}^{L-m}I\left[d\left[Y\left(i\right),Y\left(j\right)\right]\leq r\right]$$

where I[·] is the indicator function.

Sample entropy is defined as follows:

(10) $$SampEn\left(m,r\right)=-\ln\left(\frac{A}{B}\right).$$

#### 2.2.3. Permutation Entropy [18]

Permutation entropy analyzes the complexity of a time series through ordinal pattern analysis [24,25,26]. The calculation steps are as follows:

Perform phase space reconstruction on the window sequence *U*:

(11) Y(i)=[u(i),u(i+τ),…,u(i+(m−1)τ)],i=1,2,…,L−mτ+1.

Arrange the elements in each vector Y(i) in order of their values to obtain a permutation pattern $\pi_{k}$ and count the frequency $p(\pi_{k})$ of each permutation pattern $\pi_{k}$. Permutation entropy is defined as the normalized Shannon entropy:

(12) $$PermEn\left(m,\tau \right)=-\frac{1}{\ln(m!)}\sum_{k=1}^{m!}p\left(\pi_{k}\right)\ln p\left(\pi_{k}\right),\;m=3,\tau =1.$$

For each feature dimension *d* and each time window *t*, the above three types of entropy are calculated, constituting the instantaneous entropy feature sub-vector for that feature:

(13) $$e_{t}^{\left(d\right)}=\left[ApEn_{t}^{\left(d\right)},SampEn_{t}^{\left(d\right)},PermEn_{t}^{\left(d\right)}\right].$$

Simultaneously, basic statistical features are also calculated:

(14) $$s_{t}^{\left(d\right)}=\left[\mu_{t}^{\left(d\right)},\sigma_{t}^{\left(d\right)},median_{t}^{\left(d\right)}\right]$$

where $\mu_{t}^{\left(d\right)},\sigma_{t}^{\left(d\right)},median_{t}^{\left(d\right)}$ are the mean, standard deviation, and median of the feature column within the window, respectively.

Finally, the entropy features and statistical features of all features are concatenated to form the complete multimodal instantaneous entropy feature vector:

(15) $$E_{t}=\left[e_{t}^{\left(1\right)},\ldots ,e_{t}^{\left(D\right)},s_{t}^{\left(1\right)},\ldots ,s_{t}^{\left(D\right)}\right].$$

### 2.3. Dual-Branch Deep Learning Architecture

#### 2.3.1. Sequence Processing Branch

This branch processes the standardized raw sequence window $W_{t}$. We employ a multi-layer LSTM network [27,28] to capture temporal dependencies [29,30,31]. The input is the window data $S_{t}\in R^{L\times D}$ standardized by RobustScaler, and the forward calculation of the LSTM is performed:

(16) $$h_{t},\left(c_{t}\right)=LSTM\left(S_{t}\right)$$

(17) $$z_{t}^{seq}=h_{t}\left[:,-1,:\right]$$

where $z_{t}^{seq}\in R^{H}$ and the hidden state of the last time step is taken, *H* is the LSTM hidden layer dimension.

#### 2.3.2. Feature Processing Branch

This branch processes the multimodal instantaneous entropy features $E_{t}\in R^{F}$ extracted from the same window data, where *F* is the total dimension of the feature vector. Feature transformation is performed through a multilayer perceptron [32,33,34,35,36] as follows:

(18) $$h_{1}=RELU\left(W_{1}E_{t}+b_{1}\right)$$

(19) $$h_{2}=RELU\left(W_{2}h_{1}+b_{2}\right)$$

(20) $$z_{t}^{feat}=W_{3}h_{2}+b_{3}.$$

#### 2.3.3. Feature Fusion Mechanism

The outputs of the two branches are combined through concatenation fusion along the feature dimension. This approach directly merges temporal patterns from the LSTM branch with complexity dynamics from the entropy feature branch:

(21) $$z_{t}^{fusion}=\left[z_{t}^{seq};z_{t}^{feat}\right]$$

where [·;·] denotes the concatenation operation along the feature dimension. This concatenated feature vector is then passed through an output network:

(22) $$h_{3}=RELU\left(W_{4}z_{t}^{fusion}+b_{4}\right)$$

(23) $$h_{4}=RELU\left(W_{5}h_{3}+b_{5}\right)$$

(24) $${\hat{y}}_{t}=W_{6}h_{4}+b_{6}.$$

Adaptive Fusion Variant: For validation of the adaptive mechanisms, an alternative fusion approach using multi-head self-attention is implemented. In this variant, the LSTM sequence features undergo an attention-based transformation before concatenation with entropy features. Specifically, a transformer encoder layer is applied to the LSTM output sequence $h_{t}\in R^{L\times H}$ to capture cross-temporal dependencies:

(25) $$\mathrm{Attention}\left(Q,K,V\right)=\mathrm{softmax}\left(\frac{QK^{T}}{\sqrt{d_{k}}}\right)V$$

where *Q*, *K*, and *V* are query, key, and value matrices derived from the LSTM output sequence. This attention mechanism dynamically weights the importance of different temporal features based on contextual relevance. The attended sequence representation is then mean-pooled to obtain $z_{t}^{attn}\in R^{H}$, which replaces $z_{t}^{seq}$ in the concatenation operation.

### 2.4. Model Training and Evaluation Metrics

The model is optimized using the Huber loss function or the MSE loss function. The optimizer used is Adam [37] or AdamW [38], and the learning rate scheduling adopts the ReduceLROnPlateau strategy:

(26) $$\theta_{t+1}=\theta_{t}-\eta_{t}\left(\frac{\partial L}{\partial \theta }+\lambda \theta_{t}\right)$$

where $\eta_{t}$ is the adaptively adjusted learning rate, and λ is the weight decay coefficient.

For comprehensive evaluation, standard regression metrics widely used in load forecasting literature are employed:

(27) $$\mathrm{MSE}=\frac{1}{N}\sum_{i=1}^{N}{\left(y_{i}-{\hat{y}}_{i}\right)}^{2}$$

(28) $$\mathrm{MAE}=\frac{1}{N}\sum_{i=1}^{N}\left|y_{i}-{\hat{y}}_{i}\right|$$

(29) $$\mathrm{RMSE}=\sqrt{\frac{1}{N}\sum_{i=1}^{N}{\left(y_{i}-{\hat{y}}_{i}\right)}^{2}}$$

(30) $$\mathrm{MAPE}=\frac{100\%}{N}\sum_{i=1}^{N}\left|\frac{y_{i}-{\hat{y}}_{i}}{y_{i}}\right|$$

(31) $$R^{2}=1-\frac{\sum_{i=1}^{N}{\left(y_{i}-{\hat{y}}_{i}\right)}^{2}}{\sum_{i=1}^{N}{\left(y_{i}-\bar{y}\right)}^{2}}$$

where $\bar{y}$ is the mean of the true values. Additionally, for an intuitive understanding of prediction precision, the Within-Tolerance Ratio (WTR) is reported as a supplementary measure:

(32) $${\mathrm{WTR}}_{p\%}=\frac{1}{N}\sum_{i=1}^{N}I\left(\frac{|y_{i}-{\hat{y}}_{i}|}{\max(y)-\min(y)}\leq \frac{p}{100}\right)$$

(33) $$\mathrm{Weighted}\;\mathrm{WTR}=0.5\times {\mathrm{WTR}}_{5\%}+0.3\times {\mathrm{WTR}}_{10\%}+0.2\times {\mathrm{WTR}}_{15\%}$$

where I(·) is the indicator function that returns 1 if the condition is true and 0 otherwise. Weighted WTR provides an aggregated measure of prediction precision across multiple tolerance levels, while standard metrics ensure fair comparison with existing literature.

## 3. Experiments on Simulated Signals

To comprehensively evaluate the performance and characteristics of the proposed framework for power load forecasting, extensive experiments were conducted on both simulated power load data and real-world datasets [32,33,34]. This controlled experimental environment allowed systematic analysis of the framework’s predictive capabilities, parameter sensitivity, and the effectiveness of its adaptive mechanisms.

### 3.1. Simulated Dataset Description

Synthetic multimodal power load data were generated, specifically designed to validate the proposed ensemble entropy framework. The dataset contains 10,000 time points with six carefully designed feature signals and a complex target variable:

1. Chaotic Signal (Chaotic_Henon): Based on the Henon mapping with deterministic chaotic characteristics, featuring high approximate and sample entropy values suitable for validating entropy feature extraction.
2. Multifractal Signal (Multifractal_RW): Generated using weighted random walks with multi-scale characteristics, suitable for validating multi-scale entropy features.
3. Periodic Modulated Signal (Periodic_Modulated): Complex periodic signal composed of fundamental waves, modulation components, and harmonics, featuring rich spectral characteristics for spectral entropy analysis.
4. Transient Event Signal (Transient_Events): Random pulse events suitable for permutation entropy and transient feature extraction.
5. Nonlinear Oscillator Signal (Nonlinear_Oscillator): Van der Pol oscillator variant with nonlinear dynamic characteristics suitable for fuzzy entropy analysis.
6. Stochastic Volatility Signal (Stochastic_Volatility): Generated using stochastic volatility models with time-varying volatility suitable for sample entropy analysis.

Complex Target Variable Design: The target variable was constructed through nonlinear combination (tanh, sin, exp, and log transformations), time delay effects (1–5 step dependencies), cross-feature interactions, and Gaussian smoothing for continuity. This design ensures that the target exhibits complex dependencies on all input features, requiring sophisticated models for accurate prediction.

Data Partitioning: The dataset was strictly divided into training (70%), validation (15%), and test (15%) sets with no temporal overlap to prevent data leakage. Each partition maintains the same statistical characteristics to ensure fair evaluation.

Noise and Assumptions: Gaussian noise (σ=0.05×std) was added to each feature to mimic real measurement errors. The data are non-stationary and exhibit both short-term and long-term dependencies, challenging models that assume stationarity.

### 3.2. Performance of the Proposed Framework

**Figure 2:**
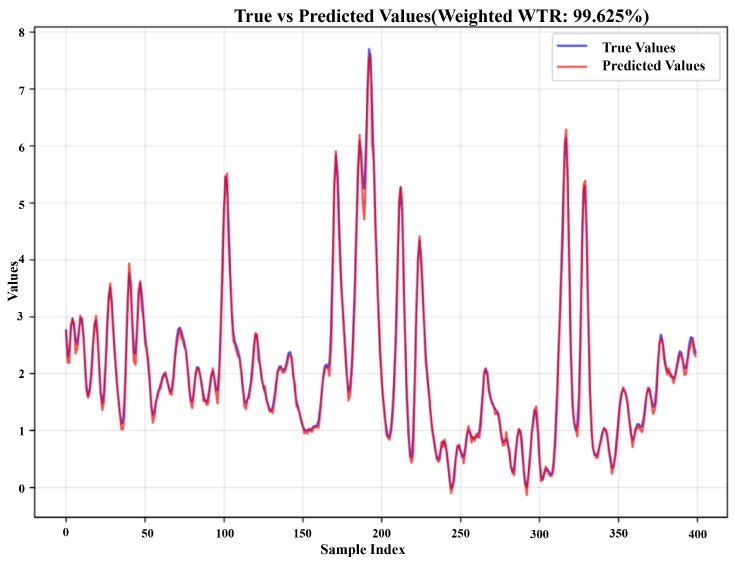
Detailed graph of weighted WTR for the simulated dataset.

The proposed framework demonstrated exceptional performance on the simulated multimodal dataset, achieving remarkable prediction accuracy and stability for short-term load forecasting. As shown in Table 1 and Figure 2, the method attained an outstanding weighted WTR of 99.625%, with complementary metrics further confirming its superior performance, MSE of 0.0125, MAE of 0.0794, RMSE of 0.1117, MAPE of 1.23%, and an R^2^ value of 0.9932. These results indicate that the framework successfully captured the complex dynamics and nonlinear relationships inherent in the multimodal simulated signals. The near-perfect R^2^ value, approaching 1.0, suggests that the framework explained virtually all the variance in the target variable, while the minimal error metrics demonstrate precise prediction capabilities. The integration of multiple instantaneous entropy features with the deep learning architecture proved particularly effective in handling the sophisticated patterns present in the simulated data, including chaotic components, periodic modulations, and transient events that characterize real-world complex systems.

### 3.3. Parameter Sensitivity Analysis

A comprehensive parameter sensitivity analysis was conducted to identify the optimal configuration for the proposed framework and understand the impact of key hyperparameters on model performance [35,36,37,38]. Four critical parameters were systematically varied: sequence length, hidden layer size, dropout rate, and learning rate, while monitoring their effects on prediction accuracy and error metrics.

**Figure 3:**
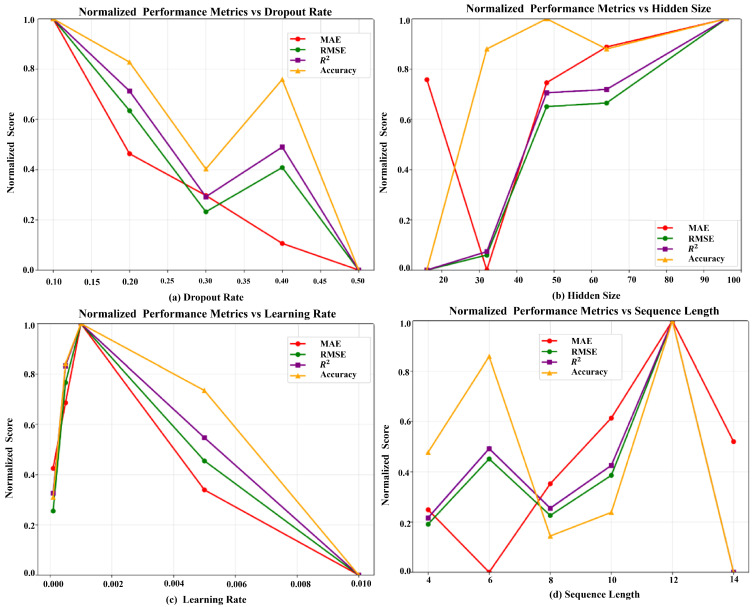
Normalized performance metrics under different parameters.

The analysis based on Figure 3 revealed that sequence length significantly influences model performance, with an optimal value of 12 time steps achieving the best balance between capturing sufficient temporal context and avoiding unnecessary complexity. At this optimal sequence length, the framework achieved a weighted WTR of 99.90% with an MSE of 0.0127. Shorter sequences (4–8 steps) failed to capture adequate temporal dependencies, while longer sequences (14 steps) introduced noise and reduced performance to 99.76% weighted WTR. For hidden layer size, the analysis demonstrated that increasing capacity generally improved performance up to 96 units, where the framework reached peak performance with 99.90% weighted WTR and MSE of 0.0110. Smaller hidden sizes (16–48 units) showed limited representational power, while the 96-unit configuration provided sufficient complexity to learn the intricate patterns in multimodal data without overfitting.

The dropout rate analysis indicated that moderate regularization (0.1) yielded optimal results, achieving 99.85% weighted WTR with an MSE of 0.0130. Higher dropout rates (0.2–0.5) progressively degraded performance due to excessive regularization, reducing weighted WTR to 99.27% at the highest dropout of 0.5. Learning rate optimization revealed that a value of 0.001 provided the best convergence characteristics, yielding 99.88% weighted WTR with an MSE of 0.0157. Both lower (0.0001) and higher (0.005–0.01) learning rates resulted in suboptimal performance, with weighted WTR dropping to 99.36% and 99.13%, respectively, due to slow convergence and overshooting issues [39].

### 3.4. Validation of Adaptive Mechanisms

**Figure 4:**
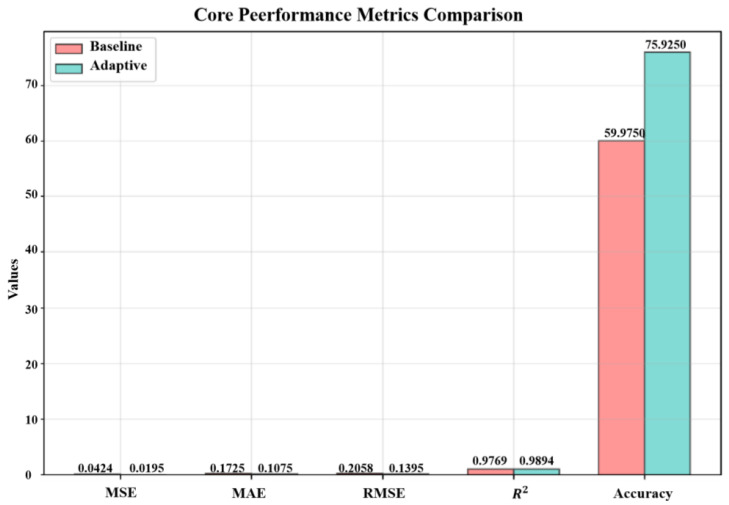
Comparison chart of the adaptive model and the baseline model.

The adaptive mechanism incorporated into the proposed framework demonstrated substantial improvements over the baseline fixed-parameter approach. Comparative analysis between the adaptive and baseline models revealed significant performance enhancements across all evaluation metrics, as summarized in Figure 4. The adaptive approach achieved 75.93% weighted WTR, representing a remarkable 26.59% absolute improvement over the baseline’s 59.98% weighted WTR. This substantial improvement was consistently reflected in error metrics, with the adaptive method reducing MSE by 54.07% (from 0.0424 to 0.0195) and MAE by 37.68% (from 0.1725 to 0.1075).

The superiority of the adaptive approach was further confirmed by additional performance indicators. The Mean Absolute Percentage Error (MAPE) decreased dramatically from 45.53% to 23.98%, while the Symmetric Mean Absolute Percentage Error (SMAPE) improved from 16.79% to 10.64%. These metrics indicate that the adaptive mechanism not only reduced absolute errors but also provided more consistent relative accuracy across different value ranges. The explained variance metric remained consistently high for both models (0.9915 for baseline vs 0.9904 for adaptive), confirming that both approaches effectively captured the underlying data patterns, with the adaptive model achieving this with substantially better precision.

Statistical significance testing provided rigorous validation of the adaptive mechanism’s superiority. Both *t*-test and Wilcoxon signed-rank test yielded extremely small *p*-values ($3.77\times 10^{-66}$ and $1.34\times 10^{-59}$, respectively), far below the conventional significance thresholds of 0.05 and 0.01. These results provide strong statistical evidence that the performance improvements achieved by the adaptive model are not due to random chance but represent genuine enhancements in predictive capability. The statistical significance of these improvements (*p*-values < $10^{-50}$) provides strong evidence that the performance gains are not attributable to random variations but reflect genuine methodological advances. The adaptive mechanism’s ability to dynamically adjust to changing signal characteristics proved particularly valuable in handling the non-stationary and transient phenomena present in the simulated multimodal signals.

## 4. Experiments on Real-World Signals

To validate the effectiveness and generalization capability of the proposed framework in real-world power systems, extensive experiments were conducted on the public ETDataset [40] for power load forecasting. This dataset represents typical challenges in power systems, including non-stationarity, multi-feature dependencies, and data scarcity issues.

In this section, through three series of experiments, the effectiveness of the proposed method on the real-world power transformer temperature dataset (ETDataset) [40] is systematically validated.

### 4.1. Performance of Multimodal Instantaneous Entropy Model

**Figure 5:**
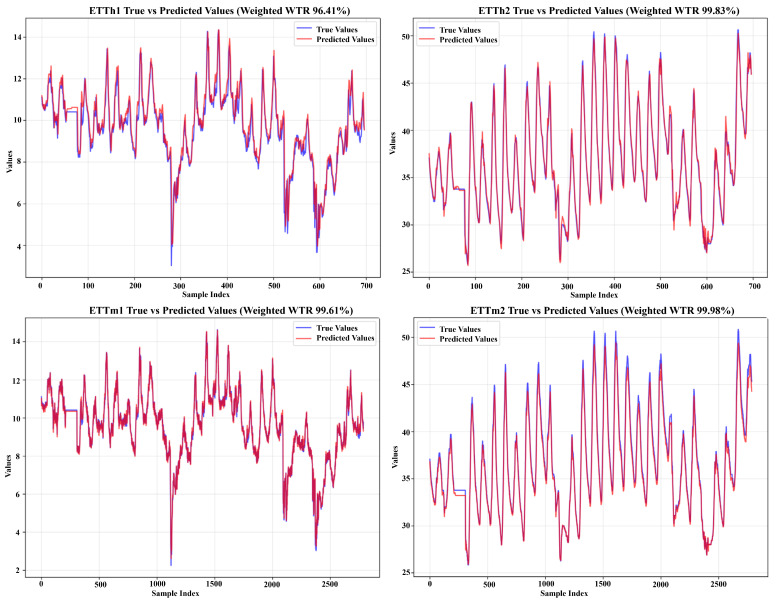
Detailed graph of weighted WTR for ETDataset.

The predictive performance of the complete framework was first evaluated on four representative datasets: ETTh1, ETTh2, ETTm1, and ETTm2. Table 2 presents the comprehensive predictive performance of the method. The results demonstrate that the framework performs excellently across all datasets. As can be seen from Figure 5, the model achieves near-perfect prediction accuracy on the ETTh2, ETTm1, and ETTm2 datasets (reaching 99.83%, 99.61%, and 99.98% weighted WTR, respectively). This indicates that for the vast majority of time points, the deviation between the framework’s predictions and the true values falls within an acceptable error margin.

Regarding the error metrics, the framework’s performance on the ETTm1 dataset is particularly outstanding, with an MSE as low as 0.073 and an MAE of 0.192, suggesting its predictions are very close to the true values. The R^2^ metric exceeds 0.9 across all datasets, reaching 0.986 on ETTm2, indicating that the framework explains 98.6% of the variance in the target variable, demonstrating strong fitting capability and explanatory power.

These excellent performance metrics prove that the ensemble entropy framework effectively captures the local dynamic characteristics of power data by fusing multiple complementary entropy measures. Combined with the synergistic use of temporal patterns from the original sequence and ensemble entropy features via the dual-branch architecture, accurate modeling of complex power load behavior is achieved through a holistic representation of system dynamics.

### 4.2. Ablation Study on ETDataset

To thoroughly evaluate the contribution of each component in the proposed framework, comprehensive ablation experiments were conducted on all four ETDataset subsets [40]. The results, presented in Table 3, clearly demonstrate the significance of the architectural innovations.

The ablation study evaluates the contribution of each component by progressively removing key elements: the complete framework with all innovations, the model without entropy features, the model with only LSTM, and the baseline MLP-only model. The baseline model serves as the fundamental reference point, representing a simple multilayer perceptron without specialized components for temporal modeling or feature extraction.

The ablation results reveal several crucial insights:

First, the complete framework achieves exceptional performance across all datasets, reaching 99% weighted WTR on three of the four subsets and 96.41% on the remaining ETTh1 dataset. This remarkable consistency underscores the robustness of the approach.

Second, the importance of ensemble entropy features is clearly demonstrated. When comparing the full framework with the “without entropy features” configuration, substantial weighted WTR improvements are observed, 19.58% on ETTh1, 23.0% on ETTh2, 25.12% on ETTm1, and 21.98% on ETTm2. These significant margins confirm that the complementary entropy ensemble captures essential dynamic patterns that traditional approaches miss.

Third, the feature fusion mechanism also contributes substantially to performance. The “LSTM+MultiModal” configuration consistently outperforms both the “LSTM Only” and baseline models across all datasets, validating the architectural design for effectively integrating temporal patterns with complexity dynamics.

Particularly noteworthy is the performance on ETTh2, where the full framework achieves 99.83% weighted WTR compared to the baseline’s 57%, representing a dramatic 42.83% improvement. This demonstrates the method’s capability to handle challenging forecasting scenarios where conventional approaches struggle.

### 4.3. Comprehensive Comparison with Baseline Models

To validate the superiority of the proposed method, extensive comparisons were conducted with several baseline models, including traditional machine learning methods and state-of-the-art deep learning architectures. For fair comparison, all models were evaluated using the same data preprocessing, training–validation–test splits, and identical hyperparameter values. The results are summarized in Table 4.

The proposed method demonstrates superior performance across all evaluation metrics compared to baseline models. The substantial improvement in error metrics can be attributed to the ensemble entropy features, which effectively capture the local dynamic complexity of power load signals. While traditional models like LSTM, TCN, and transformer focus primarily on temporal patterns, the dual-branch architecture integrates both temporal dependencies and complexity dynamics, leading to more accurate predictions.

Hyperparameter Settings: To ensure fair comparison, all models were trained with identical hyperparameter values for parameters of the same meaning. The common hyperparameters used across all models are summarized in Table 5. For model-specific architectural parameters, appropriate values were selected based on standard configurations to ensure each model performs optimally within its architectural constraints.

### 4.4. Comprehensive Comparison with State-of-the-Art Entropy Methods

An extensive comparison of the ensemble instantaneous entropy approach against five established entropy methods was conducted to validate its superiority in power load forecasting tasks. The results, summarized in Table 6, present a compelling case for the methodology.

The method demonstrates dominant performance across all evaluation metrics:

In terms of weighted WTR, the approach achieves 73.4%, significantly outperforming the second-best method (fuzzy entropy at 64.1%) by 9.3 percentage points. This substantial improvement translates to approximately 14.5% relative performance gain, highlighting the method’s superior capability in making correct predictions.

The comprehensive evaluation further confirms that the ensemble entropy framework represents a significant step forward in entropy-based time series analysis, offering substantially improved capability for capturing the complex dynamics inherent in power load data through complementary entropy measures and multi-scale characterization.

## 5. Discussion

The proposed EEADF framework addresses several limitations in contemporary STLF research. Compared to interpretable models like KANs [10], our approach focuses on enhancing predictive accuracy through ensemble entropy features. Unlike graph-based methods [11] that require predefined spatial relationships, our feature-centric approach adapts to various system configurations. While federated methods [12] address data privacy, our framework enhances local predictive accuracy and could be integrated with federated learning. Compared to quantum kernel methods [13] that leverage quantum computing for security tasks, our approach utilizes classical computing resources while achieving competitive performance through innovative ensemble entropy features.

The experimental results show that our framework outperforms traditional deep learning models. The key advantage lies in the ensemble entropy features that capture local dynamic complexity. The dual-branch architecture effectively balances global temporal patterns with local complexity, making it suitable for short-term load forecasting.

From a practical perspective, the framework can better adapt to rapid load changes, balance computational efficiency and accuracy, and generalize across different system conditions.

The ablation studies confirm the critical role of each component. The performance drop when removing entropy features or using LSTM alone underscores the necessity of combining temporal modeling with complexity dynamics.

Despite promising results, limitations include the computational cost of extracting multiple entropy features in real-time, which might limit latency-sensitive applications. Future work should optimize computational efficiency and explore integration with privacy-preserving techniques.

## 6. Conclusions

Accurate power load forecasting plays a key role in ensuring the security and economy of power systems. This paper addressed several critical challenges in STLF research by proposing the EEADF framework. Our work builds upon recent advances in interpretable models [10], spatiotemporal feature extraction [11], privacy-preserving methods [12], and quantum computing applications [13], while introducing innovative approaches to complexity characterization and feature fusion.

The proposed framework introduces three key innovations: ensemble instantaneous entropy, adaptive hierarchical fusion, and dual-branch architecture. Experimental validation on both simulated and real-world datasets demonstrates superior performance compared to baseline models and traditional entropy methods. The experimental results on both simulated and real-world datasets demonstrate competitive or superior performance relative to state-of-the-art methods.

Our work contributes to STLF research by demonstrating that ensemble entropy features can significantly enhance predictive accuracy beyond temporal patterns alone. Future research directions include optimizing computational efficiency, integrating with privacy-preserving techniques, and exploring applications in other domains.

## Figures and Tables

**Table 1:** Performance of the ensemble entropy-adaptive fusion model on simulated power load data.

MSE	MAE	RMSE	MAPE (%)	R^2^	Weighted WTR (%)
0.0125	0.0794	0.1117	1.23	0.9932	99.625

**Table 2:** Performance of the proposed method on the ETT dataset for short-term power load forecasting.

Dataset	MSE	MAE	RMSE	R^2^	Weighted WTR (%)
ETTh1	0.301	0.370	0.549	0.903	96.41
ETTh2	0.542	0.527	0.736	0.981	99.83
ETTm1	0.073	0.192	0.271	0.977	99.61
ETTm2	0.416	0.518	0.645	0.986	99.98

**Table 3:** Ablation study results on ETDataset subsets (weighted WTR, %).

Experiment Configuration	ETTh1	ETTh2	ETTm1	ETTm2
Full Model	96.41	99.83	99.61	99.98
Without Entropy Features	76.83	76.83	74.49	78.0
LSTM Only	78.0	78.0	75.66	78.0
MLP only (Baseline)	65.0	57.0	60.61	65.0

**Table 4:** Performance comparison with baseline models on the ETTh1 dataset.

Model	MSE	MAE	RMSE	MAPE (%)	R^2^	Weighted WTR (%)
Proposed Method	0.301	0.370	0.549	3.21	0.903	96.41
LSTM	8.655	2.073	2.942	15.67	0.897	78.0
TCN	12.440	2.634	3.527	18.92	0.853	75.66
Transformer	8.988	2.079	2.998	16.34	0.894	76.83
Informer	9.215	2.121	3.036	16.58	0.891	77.12

**Table 5:** Common hyperparameter settings for all models.

Hyperparameter	Value
Sequence Length	10
Learning Rate	0.001
Batch Size	16
Training Epochs	35
Weight Decay	1 × 10^−5^

**Table 6:** Performance comparison of different entropy methods.

Entropy Method	Weighted WTR (%)	Precision (%)	AUC	Recall (%)	F1-Score
Ensemble Instantaneous Entropy	73.4	68.9	0.773	88.5	0.775
Sample Entropy	60.5	61.3	0.634	50.2	0.552
Permutation Entropy	53.3	65.3	0.550	70.4	0.603
Fuzzy Entropy	64.1	59.9	0.662	86.6	0.708
Approximate Entropy	52.8	50.5	0.561	58.1	0.540

## Data Availability

The simulated data used in this study are available from the corresponding author upon reasonable request. The real-world ETDataset is publicly available at https://ojs.aaai.org/index.php/AAAI/article/view/17325 (accessed on 26 June 2025).

## Abbreviations

The following abbreviations are used in this manuscript:

MSE	Mean Squared Error
MAE	Mean Absolute Error
RMSE	Root Mean Squared Error
MAPE	Mean Absolute Percentage Error
SMAPE	Symmetric Mean Absolute Percentage Error
WTR	Within-Tolerance Ratio
LSTM	Long Short-Term Memory
MLP	Multilayer Perceptron
TCN	Temporal Convolutional Network
ApEn	Approximate Entropy
SampEn	Sample Entropy
PermEn	Permutation Entropy
ETT	Electricity Transformer Temperature (Dataset)
